# A Model to Explain How the Bacille Calmette Guérin (BCG) Vaccine Drives Interleukin-12 Production in Neonates

**DOI:** 10.1371/journal.pone.0162148

**Published:** 2016-08-29

**Authors:** Chido Loveness Kativhu, Daniel H. Libraty

**Affiliations:** Division of Infectious Diseases and Immunology, Department of Medicine, University of Massachusetts Medical School, Worcester, Massachusetts, United States of America; University of British Columbia, CANADA

## Abstract

The Bacille Calmette Guérin (BCG) vaccine is the only routine vaccination at birth that effectively induces neonatal T-helper 1 (Th1)-polarized immune responses. The primary cytokine that drives CD4+ T-cell Th1 differentiation is interleukin (IL)-12 p70, a heterodimeric cytokine composed of the IL-12 p35 and IL-12 p40 subunits. We therefore examined the mechanisms involved in BCG vaccine stimulation of IL-12 p35 and p40 production from human umbilical cord (neonatal) cells. We found that BCG bacilli did not upregulate IL-12 p35 mRNA production, but upregulated IL-12 p40 mRNA production in a Toll-like receptor (TLR)2-dependent manner, in human neonatal monocyte-derived dendritic cells (mdDCs). The combination of TLR2 signaling, Type I interferon (IFN), and Type II IFN induced maximal levels of IL-12 p35 and p40 mRNA production in human neonatal mdDCs. The cell-free supernatants of reconstituted BCG vaccine vials contained extracellular mycobacterial (BCG) DNA which could induce IFN-α (Type I IFN) production in human neonatal plasmacytoid dendritic cells (pDCs). BCG bacilli also stimulated human neonatal CD16^lo^ natural killer (NK) cells to produce IFN-γ (Type II IFN) in a TLR2-dependent manner. We have therefore proposed a model where BCG vaccine could stimulate the combination of neonatal conventional DCs (cDCs), pDCs, and CD16^lo^ NK cells to produce optimal neonatal IL-12 p35 and p40 (IL-12 p70) production and subsequent CD4+ T-cell Th1 polarization. An adjuvant that emulates the mechanism by which the BCG vaccine stimulates neonatal IL-12 p35 and p40 production could improve vaccine strategies at birth for protection against intracellular pathogens and toxins.

## Introduction

The Bacille Calmette Guérin (BCG) vaccine is given to neonates in most countries to prevent infantile tuberculous meningitis and miliary disease. It is one of the most widely used vaccines worldwide. The efficacy of neonatal BCG administration has been linked to its ability to effectively induce anti-mycobacterial CD4+ T-cell T-helper 1 (Th1)-polarized neonatal immune responses [1–5]. Neonatal BCG vaccination has also been reported to reduce neonatal and infant mortality due to diseases other than tuberculosis [6–8]. The all-cause mortality benefit of neonatal BCG vaccination may be partially related to its ability to also induce heterologous Th1-polarizing immune responses during the neonatal period [9–12]. Th1 responses are characterized by CD4+ T-cell interferon (IFN)-γ production. The primary cytokine that drives CD4+ T-cell Th1 differentiation is interleukin (IL)-12 p70, a heterodimeric cytokine composed of the IL-12 p35 and IL-12 p40 subunits [13, 14]. Neonates and infants generally have reduced IL-12 p70 production and CD4+ T-cell Th1 responses to intracellular pathogens and toxins [15–18]. The fetal and early neonatal immune system is heavily Th2 and Th17-biased [15, 17, 19, 20]. Enhanced neonatal Th1-polarized immune responses would be beneficial for combating infections with intracellular pathogens and toxin-producing organisms.

The BCG vaccine is the only routine vaccination that can be given at birth and induce Th1-polarized immune responses in neonates [1–4]. Previous reports have shown that BCG vaccination at birth results in neonatal IFN-γ production against mycobacterial antigens [1], and the levels of secreted IFN-γ are comparable to adult levels [2]. We and others have previously identified some of the heterologous immune effects of BCG vaccination, including Th1 polarization [5, 10, 11, 21]. IL-12 p70 is the prototypical Th1-polarizing cytokine [14]. We therefore examined the BCG vaccine stimulation of IL-12 p70 production from human umbilical cord (neonatal) cells by analyzing the induction of each subunit (p35 and p40). We found that BCG bacilli did not upregulate IL-12 p35 mRNA production, but upregulated IL-12 p40 mRNA production in a Toll-like receptor (TLR)2-dependent manner, in human neonatal monocyte-derived dendritic cells (mdDCs). IL-12 p35 gene transcription was previously shown to be repressed in human neonatal mdDCs [22, 23]. Goriely *et al*. [22] demonstrated that lipopolysaccharide (LPS) + IFN-γ (Type II IFN) stimulation of neonatal mdDCs was able to induce IL-12 p35 and p40 mRNA production that approached adult levels. LPS is a TLR4 ligand that stimulates both NF-κB activation and the Type I IFN pathway [24]. We found that the combination of a synthetic TLR2 agonist (NF-κB activation only), Type I IFN, and Type II IFN induced the maximal levels of IL-12 p35 and IL-12 p40 mRNA production in human neonatal mdDCs. We hypothesized that the BCG vaccine uses the same signaling combination, utilizing multiple innate immune cells, in order to produce adult-like levels of IL-12 p35 and p40 (IL-12 p70) in neonates. We found that the cell-free supernatants of reconstituted BCG vaccine vials contained extracellular mycobacterial (BCG) DNA which could induce IFN-α (Type I IFN) production in human neonatal plasmacytoid dendritic cells (pDCs). We also found that BCG bacilli stimulated human neonatal CD16^lo^ natural killer (NK) cells to produce IFN-γ (Type II IFN) in a TLR2-dependent manner. We have therefore proposed a model where BCG vaccine could stimulate the combination of neonatal conventional DCs (cDCs), pDCs, and CD16^lo^ NK cells thereby producing optimal neonatal IL-12 p35 and p40 production (IL-12 p70) and subsequent CD4+ T-cell Th1 polarization.

## Methods

### Ethics Statement

Human umbilical cord blood was collected from the full-term placentas of healthy mothers with uncomplicated deliveries at the University of Massachusetts Memorial Medical Center Labor and Delivery Ward. The clinical study protocol was approved by the University of Massachusetts Medical School (UMMS) institutional review board (IRB). Umbilical cord blood was collected after verbal consent was obtained. Verbal consent was approved by the UMMS IRB, as obtaining written consent was not feasible on the Labor and Delivery Ward. Subject’s verbal consent was recorded on a datasheet.

### Drugs, reagents, and antibodies

The recombinant (r) cytokines IL-2, IL-3, IL-4 and GM-CSF were obtained from Peprotech Inc., and used at final concentrations of 100 U/ml (rIL-2), 1000 U/ml (rIL-3), 100 U/ml (rIL-4) and 800 U/ml (rGM-CSF). The monoclonal antibodies (mAbs) against human TLR2 (clone #383936) and the IgG_2B_ isotype control (clone #20116) were obtained from R&D Systems, and used at a final concentration of 20 μg/ml. Unless otherwise stated, all mAbs for FACS staining were obtained from BD Biosciences. For cell stimulations (mdDCs, pDCs, and NK cells), the Tice BCG vaccine (Merck) was reconstituted in sterile water, per the manufacturer’s instructions, and used at a final concentration of 8x10^4^ colony forming units (cfu)/ml. For the pDC experiments, the synthetic TLR2 agonist Pam3CSK4 (Invivogen), was used at a concentration of 300 ng/ml; recombinant human IFN-β (PBL Assay Science) was used at a concentration of 1x10^4^ U/ml; and recombinant human IFN-γ (PBL Assay Science) was used at a concentration of 1x10^3^ U/ml.

### Cells

Cord blood mononuclear cells (CBMC) were isolated by density gradient centrifugation (Histopaque™, Sigma-Aldrich) and cryopreserved within 24 hours post-delivery. To generate mdDCs, CBMC CD14+ monocytes were negatively selected by MACS® using the Monocyte Isolation kit II (Miltenyi Biotec). The monocytes were then plated in 24-well plates at 1.7x10^6^ cells/well, and cultured in RPMI 1640/10% FCS/rIL-4/rGM-CSF for 7 days. Differentiation into immature mdDCs (lineage^-^CD1c^+^CD83^lo^) was ≥ 70%, as confirmed by flow cytometry. pDCs were isolated from CBMC by MACS® negative selection using the Plasmacytoid Dendritic Cell Isolation Kit II (Miltenyi Biotec), and cultured in RPMI 1640/10% FCS/rIL-3 (>95% purity). NK cells were negatively selected by MACS® using the NK Cell Isolation Kit Human (Miltenyi Biotec), and cultured in RPMI 1640/10% FCS/rIL-2 (>95% purity).

### BCG stimulation of mdDCs

Immature mdDCs were left unstimulated, or stimulated with BCG x 18–24 hours ± pre-incubation for 1 hour with a TLR2 blocking mAb or an isotype control mAb. An approximate multiplicity of infection (MOI) = 5 was used. The cells were then lysed and cytosolic RNA was extracted using RNeasy Plus (Qiagen), according to the manufacturer’s instructions. Relative gene expression for IL-12 p35 and IL-12 p40 mRNA was determined by quantitative (q)RT-qPCR. GAPDH was used as the housekeeping gene.

### BCG DNA stimulation of pDCs

BCG DNA was extracted from a BCG culture (kindly provided by Christopher Sassetti, UMMS). pDCs were stimulated with 100 μg/ml BCG DNA for 8 hours; cell lysates were collected for IFN-α1 and IFN-α2 gene expression analysis using qRT-PCR. β-actin was used as the housekeeping gene. In some experiments, pDCs were stimulated with intact BCG bacilli (Tice strain) in a similar fashion as described for mdDCs. All qRT-qPCR assays were performed using the TaqMan Gene Expression assay system (Applied Biosystems), following the manufacturer’s instructions. Analysis was performed using ABI 7500 Software v2.0 (Applied Biosystems).

### Identifying extracellular BCG DNA in BCG vaccine vials

BCG vaccine vials were obtained from two different manufacturers- Tice BCG (Pasteur strain) from Merck, USA, and Tokyo BCG (Japan strain) from the Japan BCG Laboratory, Japan. The BCG vaccine vials were reconstituted in sterile water, per the manufacturer’s instructions, and spun down to collect the cell-free supernatants for PCR. PCR for 2 mycobacterial genes, *rpo*B and the RD8 portion in BCG, was performed as described in [25].

### Flow cytometry

Cord blood NK cells were isolated by MACS®, and seeded at a density of 1x10^6^ cells/tube. The cells were stimulated with BCG overnight, incubated with Brefeldin-A (1 μg/ml, Qiagen) for the last 4 hours, stained with a vital dye (L/D Aqua), fixed and permeabilized (Cytofix/Cytoperm, BD Biosciences), and then stained with the mouse anti-human mAbs to CD3 (clone SK7), CD16 (clone 3G8), CD56 (clone NCAM16.2), and IFN-γ (clone 25723.11). A minimum of 50,000 events were acquired. To test if IFN-γ production was dependent on TLR2 signaling, isolated NK cells were pre-incubated for 1 hour with either anti-TLR2 antibody or IgG isotype control (R&D Systems) prior to stimulation with BCG. Events were acquired on an LSRII flow cytometer (Becton Dickinson), and analyzed using FlowJo™ v10.1 software (FlowJo LLC).

### Statistical analysis

Statistical analysis was done using Prism 7 software (GraphPad Prism 7). Comparisons between paired groups were made using the non-parametric Wilcoxon's signed-rank test. P-values < 0.05 were considered significant.

## Results

### BCG does not induce IL-12 p35 mRNA production, but induces IL-12 p40 mRNA production in a TLR2-dependent manner, from human neonatal mdDCs

**Fig 1:**
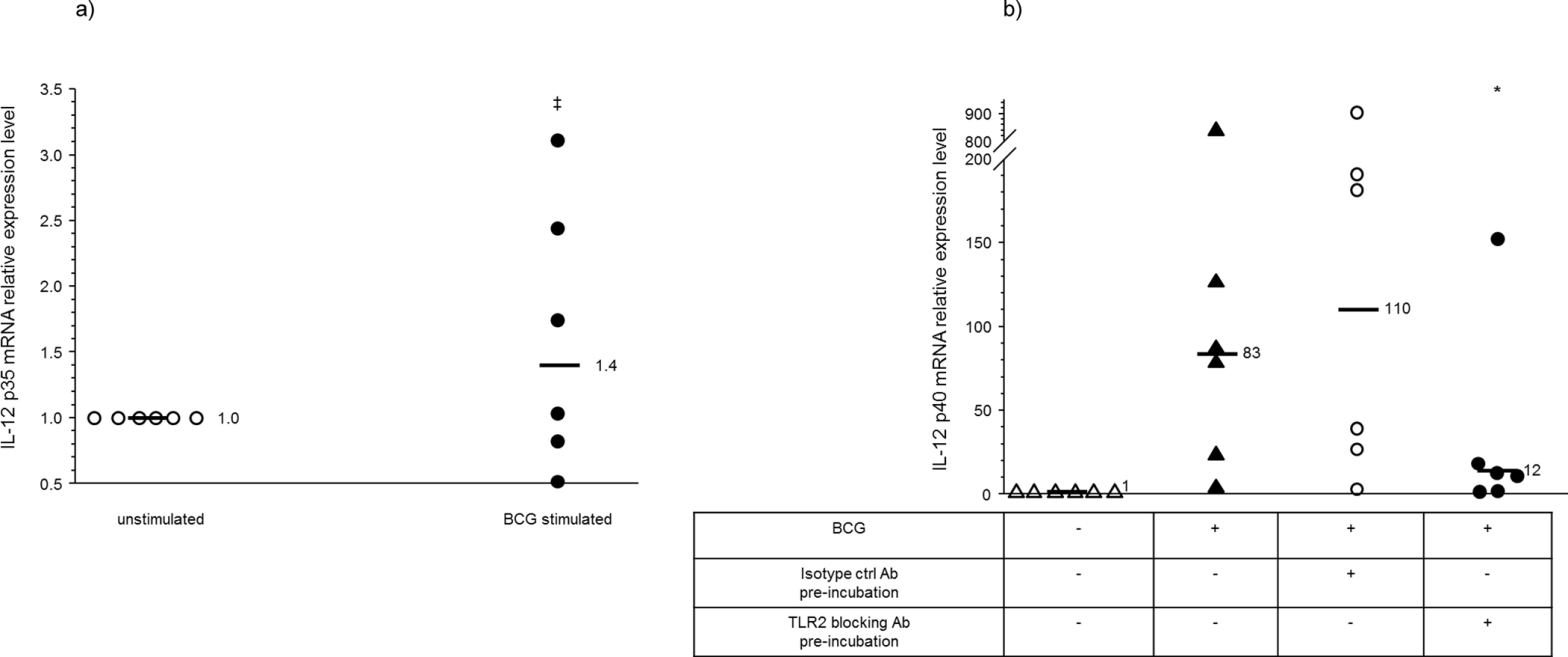
Bacille Calmette Guérin (BCG) does not induce IL-12 p35 mRNA production from human neonatal monocyte-derived dendritic cells (mdDCs), but induces IL-12 p40 mRNA production in a Toll-like receptor (TLR)2-dependent manner. Human umbilical cord blood CD14+ monocytes were differentiated into mdDCs using rIL-4 and rGM-CSF, stimulated with BCG x 18–24 hours, and then cellular mRNA was isolated for qRT-PCR. (a) relative expression of IL-12 p35 mRNA levels upon BCG stimulation compared to unstimulated control. Bars are median values, ^‡^ p = 0.3, *n* = 6 independent experiments; (b) relative expression of IL-12 p40 mRNA levels upon BCG stimulation compared to unstimulated control, in the presence or absence of a TLR2 blocking antibody. Bars are median values, * p<0.05 compared to BCG stimulation with isotype control antibody pre-incubation, *n* = 6 independent experiments.

It has been well established that the BCG vaccine is able to produce anti-mycobacterial neonatal CD4+ T-cell Th1 responses *in vivo* [1–5]. The prototypical Th1-polarizing cytokine is IL-12 p70 [14]. IL-12 p70 is a heterodimeric cytokine made up of the p35 and p40 subunits. We therefore examined BCG stimulation of IL-12 p35 and p40 mRNA production in human neonatal mdDCs. Human umbilical cord blood CD14+ monocytes were differentiated into mdDCs using IL-4 and GM-CSF. We found that BCG stimulation did not upregulate IL-12 p35 mRNA production in human neonatal mdDCs (Fig 1A). However, BCG upregulated IL-12 p40 mRNA production in a TLR2-dependent manner (Fig 1B). The addition of TLR4 blockade to TLR2 blocking did not further diminish IL-12 p40 mRNA production (data not shown).

### The combination of a synthetic TLR2 agonist (Pam3CSK4), rIFN-β (Type I IFN), and rIFN-γ (Type II IFN) stimulates maximal levels of IL-12 p35 and IL-12 p40 mRNA production in human neonatal mdDCs

**Fig 2:**
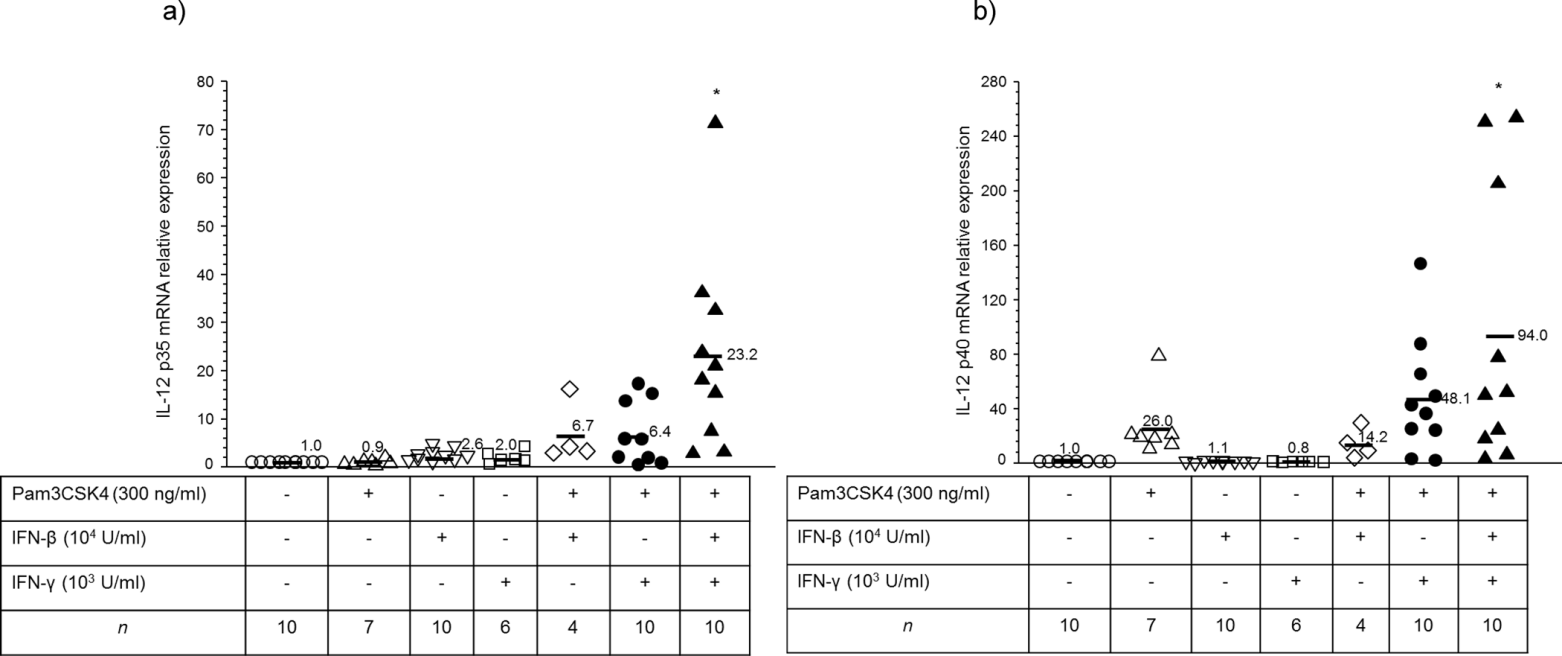
Optimization of interleukin (IL)-12 p35 and p40 mRNA expression in human neonatal monocyte-derived dendritic cells (mdDCs). Neonatal mdDCs were primed with recombinant (r) interferon-β (IFN-β) (Type I IFN), and rIFN-γ (Type II IFN) for 12 hours prior to stimulating them with a synthetic TLR2 agonist, Pam3CSK4. The combination of Pam3CSK4, rIFN-β, and rIFN-γ induced the maximal levels of (a) interleukin (IL)-12 p35 mRNA and (b) IL-12 p40 mRNA expression in human neonatal monocyte-derived dendritic cells (mdDCs). * p<0.05 compared to unstimulated control. Data points represent individual donors, the number of individual donors for each condition is shown in the figure, and bars are mean values.

Human neonatal mdDCs have repressed IL-12 p35 gene expression compared to adult cells [22]. The transcriptional repression of IL-12 p35 in human neonatal mdDCs takes place at the chromatin level [23]. We interpreted data shown by Goriely *et al*. [22], that LPS + IFN-γ could increase IL-12 p35 gene expression in human neonatal mdDCs in similar fashion to adult mdDCs, to mean that a combination of NF-κB activation, Type I IFN signaling, and Type II IFN priming was needed for optimal neonatal IL-12 p35 production. We therefore primed neonatal mdDCs for 12 h with IFN-β and IFN-γ prior to stimulating them with a synthetic TLR2 agonist, Pam3CSK4. We observed that the combination of Pam3CSK4 + rIFN-β + rIFN-γ induced the maximal levels of IL-12 p35 mRNA (Fig 2A) and IL-12 p40 mRNA production (Fig 2B) from human neonatal mdDCs, and the mRNA relative expression levels were normally distributed (SPSS Statistics v24.0).

### The BCG vaccine contains extracellular mycobacterial (BCG) DNA which can induce IFN-α production in human neonatal pDCs

**Fig 3:**
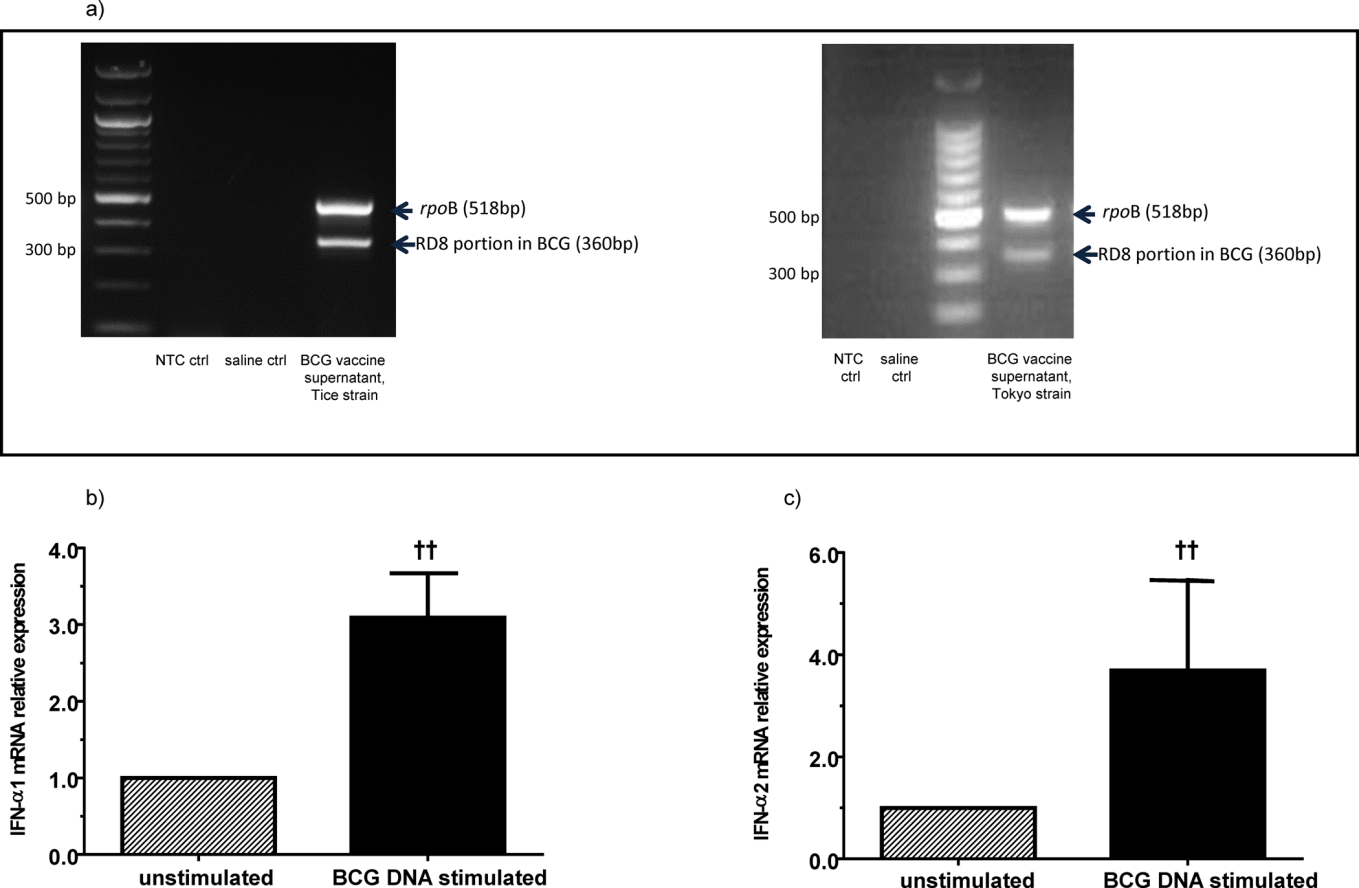
The Bacille Calmette Guérin (BCG) vaccine contains extracellular mycobacterial (BCG) DNA which induces Type I interferon (IFN) production in human neonatal plasmacytoid dendritic cells (pDCs). (a) The cell-free supernatants of reconstituted BCG vaccine vials from two different manufacturers contain BCG DNA. PCR for 2 mycobacterial (BCG-specific) genes on the cell-free supernatants was performed as described in the Methods section, and the PCR products were run on an agarose gel and stained with ethidium bromide. Isolated human umbilical cord blood pDCs were stimulated with BCG DNA (100 μg/ml) for 8 hours and the relative expression levels of (b) IFN-α1 mRNA and (c) IFN-α2 mRNA were determined by qRT-PCR. ^††^ p = 0.07, *n* = 3 independent experiments, bars are median values and error bars are S.D.

pDCs are the predominant cellular source of secreted Type I IFN (IFN-α) [26]. We found that BCG bacilli did not stimulate IFN-α production from human neonatal pDCs (data not shown). However, using PCR for 2 genes used to identify BCG (*rpo*B and the RD8 portion in BCG) [25], we found that there was extracellular mycobacterial (BCG) DNA in the cell-free supernatants of reconstituted BCG vaccine vials (Fig 3A). BCG DNA stimulation induced modest upregulation of IFN-α1&2 mRNA production in isolated human umbilical cord pDCs at 8 hours (Fig 3B & 3C). Time points at 8, 16, and 24 hours were examined, and the greatest upregulation was seen at 8 hours. IFN-α is the secreted form of Type I IFN from pDCs [26], and IFN-α and IFN-β are both Type I IFNs that signal through the Type I IFN receptor [27].

### BCG bacilli stimulate human neonatal CD16^lo^ NK cells to produce IFN-γ in a TLR2-dependent manner

**Fig 4:**
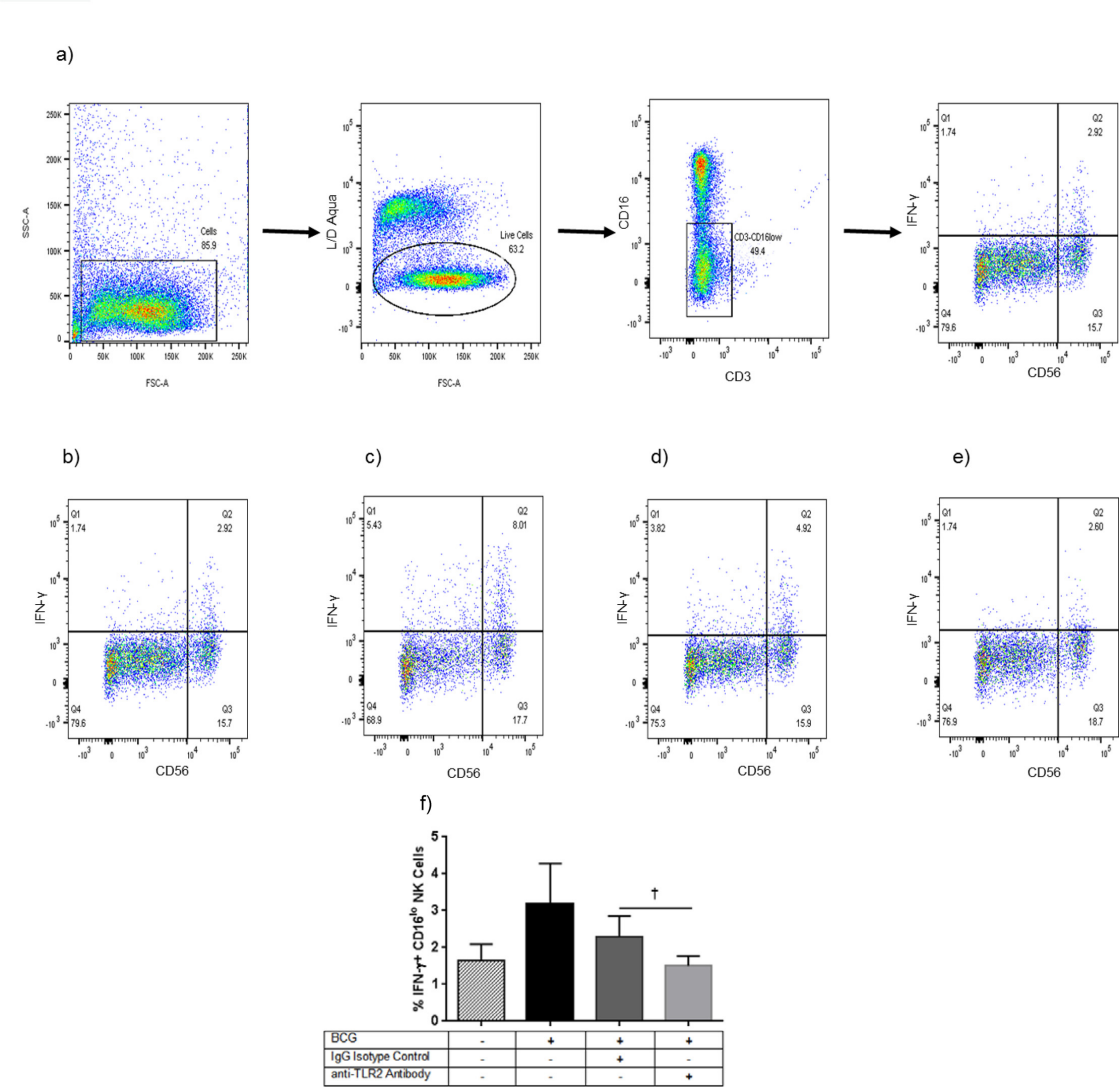
BCG stimulates CD16^lo^ natural killer (NK) cells to produce interferon (IFN)-γ in a TLR2-dependent manner. Human neonatal NK cells were isolated from umbilical cord blood, stimulated with Bacille Calmette Guérin (BCG) overnight (in the presence of Brefeldin-A for the last 4 hours), stained with a vital dye (L/D Aqua), and intracellularly stained for CD3, CD16, CD56, and interferon (IFN)-γ for flow cytometry analysis. In Fig 4a-e, one representative experiment is shown. (a) gating strategy, (b) unstimulated control, (c) BCG stimulated, (d) BCG stimulated and pre-incubated with an isotype control antibody, (e) BCG stimulated and pre-incubated with a Toll-like receptor (TLR)2 blocking antibody. (f) Summary data for *n* = 5 independent experiments. Bars are mean values and error bars are S.D. ^†^ p = 0.06, BCG+TLR2 blocking IgG vs. BCG+isotype control IgG.

NK cells are a source of early IFN-γ production, and they express TLR2 [28]. Isolated human umbilical cord NK cells were stimulated with BCG and intracellularly stained for IFN-γ production. BCG stimulation induced IFN-γ production from CD16^lo^ NK cells. NK cells pre-incubated with an anti-TLR2 antibody showed diminished IFN-γ production compared to IgG isotype control (Fig 4).

## Discussion

**Fig 5:**
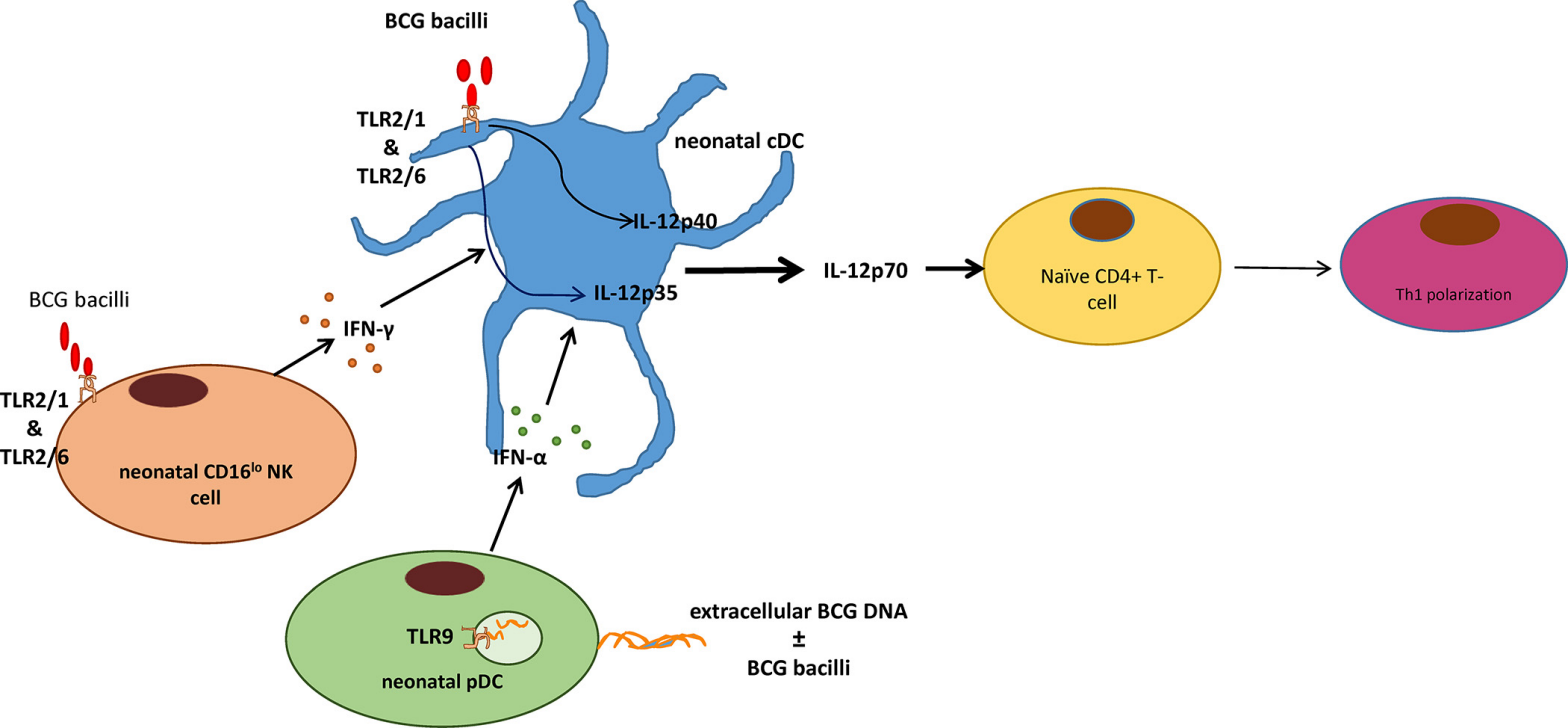
Illustrative model demonstrating how the Bacille Calmette Guérin (BCG) vaccine might drive interleukin (IL)-12 p35 and p40 (IL-12 p70) production and CD4+ T-cell T-helper 1 (Th1) polarization in neonates. IFN = interferon; cDC = conventional dendritic cell; NK = natural killer; pDC = plasmacytoid dendritic cell; TLR = Toll-like receptor.

The BCG vaccine is unique in its ability to induce Th1-polarized immune responses during the neonatal period. We propose that the BCG vaccine induces Th1 polarization in neonates by producing adult-like levels of IL-12 p35 and p40 (IL-12 p70) from cDCs, the predominant source of IL-12. BCG bacilli could activate TLR2 signaling in cDCs and CD16^lo^ NK cells driving IL-12 p40 gene expression and IFN-γ secretion, respectively. Extracellular mycobacterial DNA in the BCG vaccine also could activate TLR9 signaling in pDCs driving IFN-α secretion. The combination of direct TLR2 activation on cDCs, TLR9-mediated IFN-α secretion by pDCs, and TLR2-mediated IFN-γ secretion by CD16^lo^ NK cells, could act in concert to produce adult-like levels of IL-12 p35 and p40 mRNAs in the neonatal mdDCs. The adult-like levels of IL-12 p70 (IL-12 p35 and p40) from the cDCs then would drive Th1 differentiation of naïve CD4+ T-cells. An illustrative model is shown in Fig 5.

The mycobacterial cell wall contains numerous lipoproteins and glycolipoproteins that act as potent TLR2/1 and TLR2/6 ligands [29–34]. The predominant role of TLR2 activation and signaling that we observed in BCG-induced IL-12 p40 production from human neonatal mdDCs is consistent with a previous publication utilizing TLR2 knockout mice [35]. Unlike another study using TLR4 knockout mice [36], we found that blocking TLR4 did not have an additive effect on the TLR2 blocking for BCG-stimulated IL-12 p40 production in human neonatal mdDCs. Given the predominant effect of TLR2 blockade, it would have been difficult to observe an additional effect of TLR4 blockade. Human neonatal mdDCs have repressed IL-12 p35 gene expression compared to adult cells [22]. Although TLR2 stimulation by itself (NF-κB activation only) was sufficient to upregulate IL-12 p40 mRNA a little, it was not sufficient to upregulate IL-12 p35 mRNA production in human neonatal mdDCs. TLR2 stimulation + Type II IFN could upregulate IL-12 p35 mRNA in neonatal mdDCs to a degree, but the combination of TLR2 stimulation + Type I IFN + Type II IFN achieved the maximal upregulation of IL-12 p35 and p40 mRNAs. In the cord blood (neonatal) mdDC preparations, the vast majority of the non-mdDCs were undifferentiated monocytes (approximately 25–30%). Although mdDCs are a primary source of IL-12, we cannot exclude the possibility that monocyte-derived IL-12 p35 and p40 production contributed to our findings. IL-12 p70 protein levels were also not examined in these experiments, as the upregulation solely of IL-12 p40 would manifest as increased IL-12 p70 levels. The absence of IL-12 p35 and p40 protein measurements is a potential limitation of this study.

pDCs are the predominant cellular source of secreted Type I IFN (IFN-α) largely through TLR7/9 activation [26]. Mycobacterial DNA is a well-recognized potent TLR9 activating ligand [37]. We found that the cell-free supernatants of reconstituted BCG vaccine vials from 2 different manufacturers contained naked extracellular BCG DNA, and BCG DNA could stimulate IFN-α production from human neonatal pDCs (likely through TLR9 activation). The naked extracellular mycobacterial DNA in BCG vaccine vials is likely the result of dead and lysed BCG bacilli from the BCG vaccine manufacturing process. It should be noted that quality control standards established by the World Health Organization (WHO) for BCG vaccine products do not assess the presence of mycobacterial DNA in reconstituted vaccines [38]. The presence of extracellular mycobacterial DNA in reconstituted BCG vaccine vials likely serves as a TLR9 adjuvant, and an *in vivo* stimulus of local neonatal pDC Type I IFN production when the vaccine is given at birth. Mycobacterial DNA in the intact BCG bacilli might also contribute to TLR9-mediated neonatal pDC IFN-α production. However, we also observed that intact BCG bacilli did not stimulate IFN-α production from human neonatal pDCs. It is likely that the DNA in intact BCG bacilli cannot efficiently access TLR9 (in an endosomal location) in the pDCs. In addition to its effects on IL-12 p35 and p40 upregulation in neonates, Type I IFN can polarize neonatal CD4+ T-cells to a Th1 phenotype in an IL-12 independent manner [39]. Type I IFN can also induce IFN-γ production from neonatal NK cells [40].

Finally, NK cells are a source of early IFN-γ (Type II IFN) production and express TLR2 [41, 42]. CBMCs have a lower percentage of CD56^+^ NK cells compared to adult peripheral blood mononuclear cells (PBMCs) [43]. Confirming a previous study by Watkins *et al*.[44], we found that BCG bacilli could stimulate neonatal NK cells to produce IFN-γ. We further demonstrated that BCG bacilli induce IFN-γ production specifically from the CD16^lo^ subset of human neonatal NK cells in a TLR2-dependent manner. The purity of the neonatal NK cell preparation (>95%) and the TLR2-dependence suggest that BCG bacilli are directly stimulating IFN-γ production in the CD16^lo^ NK cells. However, we cannot exclude the possibility of some indirect NK cell activation.

Combining the aforementioned data, we have proposed a model where the BCG vaccine could stimulate a combination of TLRs 2&9 signaling, and Types I and II IFN production, among neonatal cDCs, pDCs, and CD16^lo^ NK cells. Intact BCG bacilli have TLR2 ligands in their cell wall, and extracellular BCG DNA in the vaccine vials could act as a pDC TLR9 ligand. The combination of the signals and cell subsets noted above could act in concert to optimize neonatal IL-12 p35 and p40 (IL-12 p70) production and subsequent CD4+ T-cell Th1 polarization in neonates (Fig 5). We postulate that the interaction between neonatal cDCs, pDCs, and CD16^lo^ NK cells would take place within the dermis and regional draining lymph nodes of the BCG vaccination site *in vivo*.

Our data demonstrates the potential mechanism by which the BCG vaccine could stimulate optimal neonatal IL-12 p35 and p40 (IL-12 p70) production by utilizing multiple innate immune cells. The production of adult-like levels of IL-12 p70 in neonates would promote the anti-mycobacterial CD4+ T-cell Th1 polarization that has been seen following neonatal BCG vaccination, and it could also contribute to beneficial heterologous Th1 immune effects of BCG vaccination [5, 9–11, 21]. Developing an effective Th1-inducing adjuvant for the neonatal period would be the first step towards the goal of improving vaccinations against toxins (*e*.*g*. diphtheria/pertussis/tetanus) and viruses (*e*.*g*. polioviruses, measles virus) so that they could begin at birth and require fewer booster doses. Our data suggests that an effective neonatal Th1-inducing adjuvant should be comprised of a TLR2 agonist, Type I IFN, and Type II IFN.
